# 

**Published:** 1880-02

**Authors:** 


### CONTENTS.

### COMMUNICATIONS.

Cancroid Stomatitis........ 57

Tobacco ........ 61

Report of the Committee of Dental Chemistry ........ 66

Professional Ethics........ 70

Dental Education ........ 78

Mechanical Dentistry ........ 85

SCIENTIFIC NEWS ........ 89

Miscellaneous Notes ........ 91

EDITORIAL........ 94

